# Cumulative excess weight exposure over time and cardiovascular risk: A prospective cohort study

**DOI:** 10.1371/journal.pone.0344620

**Published:** 2026-04-08

**Authors:** Alexander Turchin, Maria Shubina, Fritha J. Morrison, Nadia N. Ahmad, Lisa M. Neff, Hong Kan

**Affiliations:** 1 Brigham and Women’s Hospital, Boston, Massachusetts, United States of America; 2 Harvard Medical School, Boston, Massachusetts, United States of America; 3 Eli Lilly and Company, Indianapolis, Indiana, United States of America; The Chinese University of Hong Kong, HONG KONG

## Abstract

**Background:**

Obesity at a given point in time is a known cardiovascular risk factor. However, the contribution of long-term excess weight exposure to cardiovascular risk has not been well established. We therefore conducted a study to evaluate the relationship between long-term excess weight exposure and incidence of cardiovascular events.

**Methods:**

This secondary analysis of a prospective cohort study analyzed data from adult participants in Nurses’ Health Study and Health Professionals Follow-Up Study with a BMI > 25 kg/m^2^ between 01/01/1990 and 12/31/1999. We investigated the relationship between cumulative exposure to body mass index (BMI) > 25 kg/m^2^ between 1990 and 1999 and time to the composite primary outcome of fatal and non-fatal myocardial infarction (MI) and stroke beginning in 2000.

**Results:**

The mean baseline BMI of 136,498 study participants was 27.2 kg/m^2^ and the mean annualized cumulative excess BMI exposure was 3.9 kg/m^2^. In multivariable analysis having annualized cumulative excess BMI in the 4^th^ vs. 1^st^ quartiles was associated with an increased cardiovascular risk for women aged < 35 (HR 1.60; 95% CI 1.05–2.44) and 35−50 years (HR 1.27; 95% CI 1.01–1.58); and for men aged 35−50 (HR 1.57; 95% CI 1.22–2.03) and 50−65 (HR 1.23; 95% CI 1.02–1.48). There was no increase in cardiovascular risk with greater excess BMI exposure for women older than 50 and for men older than 65. Baseline BMI (in 1990) was not associated with cardiovascular risk in models adjusted for cumulative excess BMI exposure.

**Conclusion:**

Long-term excess weight exposure plays a greater role in cardiovascular risk than weight at a single point in time. This risk is strongest in younger individuals.

## Introduction

Prevalence of overweight and obesity is growing in the U.S. and worldwide [1–3]. This creates a significant public health challenge as obesity is associated with a broad swathe of adverse clinical outcomes, ranging from sleep apnea to liver disease to cancer and many others [4–8]. Importantly, individuals with overweight or obesity are known to have an increased risk of cardiovascular events [9,10] – a leading cause of death [11].

Most previously published investigations have only examined the relationship of cardiovascular risk with measures of obesity taken at a single point in time. However, other studies indicate that measures of longitudinal exposure to cardiovascular risk factors are an important independent predictor of long-term incidence of cardiovascular events [12]. Previously conducted research of long-term exposure to obesity and cardiovascular risk was limited by a constrained age range and smaller number of participants [13]. We have therefore conducted a study that leveraged a large prospectively collected nationwide dataset to investigate cumulative exposure to excess weight and long-term incidence of cardiovascular events.

## Methods

### Study design

We analyzed data from a prospective cohort study to investigate the relationship between exposure to body mass index (BMI) > 25 kg/m^2^ over a decade and subsequent incidence of cardiovascular events.

### Study cohort

This study included Nurses’ Health Study (NHS) and Health Professionals Follow-Up Study (HPFS) participants who had at least one BMI measurement > 25 kg/m^2^ between 01/01/1990 and 12/31/1999 and were followed into the outcome ascertainment period (on or after 01/01/2000). Threshold of 25 kg/m^2^ was chosen because, particularly in younger individuals, increase in development of cardiovascular risk factors and incidence of cardiovascular events with greater weight can already be seen in this BMI category [14,15]. Participants with missing baseline BMI (prior to 01/01/1990) were excluded from the analysis. NHS I cohort was recruited in 1976 and consisted of 121,700 female nurses aged 30–55 years. NHS II cohort was recruited in 1989 and consisted of 116,429 female nurses aged 25–42 years. The HPFS cohort was established in 1986, when 51,529 male US health professionals (dentists, optometrists, osteopaths, podiatrists, pharmacists, and veterinarians) aged 4075 years were recruited. Since their recruitment, participants have been surveyed every two years, assessing lifestyle and health status with validated, self-administered questionnaires. Detailed information on recruitment and follow-up procedures of the NHS and HPFS has been published [16,17].

### Study measurements

Study participants’ baseline demographic characteristics, vital signs, lifestyle characteristics and comorbidities were ascertained using their NHS and HPFS information at study entry on 01/01/1990. Annualized cumulative excess BMI exposure served as the primary predictor variable. It was calculated as area under the curve of BMI above 25 kg/m^2^ between 01/01/1990 and 12/31/1999 divided by 10. In other words, excess BMI accumulation was not modeled as a time-varying covariate but rather as fixed variable calculated over a decade prior to the beginning of follow-up to evaluate study outcomes.

The primary endpoint for the study was the composite endpoint of fatal and non-fatal myocardial infarction (MI) or cerebrovascular accident (CVA). Incidence of the primary endpoint was ascertained beginning on 01/01/2000 (outcome ascertainment period). Non-fatal MI and CVA events were confirmed by manual review of participants’ medical records according to World Health Organization criteria (for MI) [18] and National Stroke Criteria (for CVA) [19]. Fatal MI or stroke were identified based on the cause of death listed on the death certificate.

### Statistical analysis

Descriptive statistics were calculated using frequency counts for categorical and binary variables and summary statistics including mean, standard deviation, and median for continuous variables.

Cox multivariable proportional hazards models were used to evaluate the relationship between annualized cumulative excess BMI exposure and the composite primary outcome. Patient demographics (sex, age, race/ ethnicity); smoking status; baseline BMI (as of 01/01/1990); history of diabetes, heart failure, hypertension or atherosclerotic cardiovascular disease (ASCVD); and family history of diabetes or ASCVD were included as covariates in the analysis. Models were stratified by sex and age (< 35; 35–49.9; 50–64.9; ≥ 65 years old). Models were constructed using Fine-Gray subdistribution proportional hazards models [20] with death from any condition not included in the primary composite endpoint as a competing risk event. The proportional hazards assumption was assessed by visual inspection of cumulative incidence curves for the primary outcome grouped by quartiles of the excess BMI variable. Analyses were conducted using SAS 9.4 (Cary, NC).

### Ethical considerations

This study was approved by the Mass General Brigham institutional review board, and the requirement for informed consent was waived.

### Role of the funding sources

This study was supported in part by Eli Lilly and Company and the National Institutes of Health (UM1 CA186107; R01 HL034594; R01 HL088521; U01 CA176726; U01 HL145386; and U01 CA 167552). NIH had no involvement in the analysis or the decision to publish the results. Employees of Eli Lilly were involved in the study design, interpretation of data and writing the manuscript. The content does not necessarily represent the official views of the National Institutes of Health.

## Results

### Study cohort

**Fig 1:**
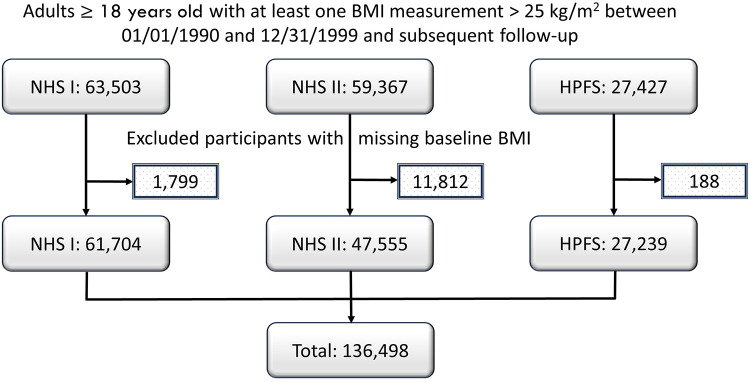
Participant Flow Chart.

**Fig 2:**
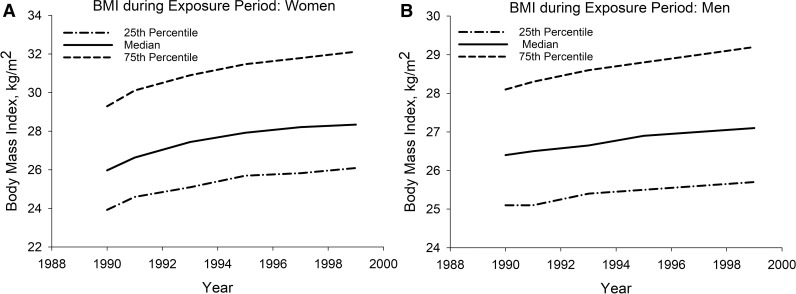
BMI Changes of Study Participants during the Exposure Period. **A.** Women. **B.** Men.

We identified a total of 150,297 NHS and HPFS study participants with at least one BMI measurement > 25 kg/m^2^ between 01/01/1990 and 12/31/1999 and with subsequent follow-up. After 13,799 participants without a baseline BMI measurement were excluded, 136,498 participants were included in the analysis (Fig 1). A total of 6,862 (5.0%) study participants had baseline ASCVD and 3,587 (2.6%) had diabetes. Age of the study participants ranged from 25 to 69 years for women and from 43 to 80 years for men. Their mean baseline BMI was 27.2 (SD 4.6) kg/m^2^ (Table 1). Their mean annualized cumulative excess BMI over 25 kg/m^2^ between 1990 and 1999 (Table 2) was 3.9 (SD 4.4) kg/m^2^ and was higher for women than for men (4.2 vs. 2.6 kg/m^2^ per year; p < 0.001). Compared to baseline, BMI at the end of follow-up increased by at least 1.0 kg/m^2^ for 88,488 (64.8%) and decreased by at least 1.0 kg/m^2^ for 15,926 (11.7%) study participants (Fig 2).

**Table 1:** Baseline Characteristics of Study Participants.

Characteristic	Women	Men	P-value
Total population, n	109,259	27,239	
Age, mean (SD)	46.8 (11.8)	56.2 (8.9)	<.001
Baseline BMI, kg/m^2^, mean (SD)	27.3 (4.9)	26.9 (2.9)	<.001
Systolic blood pressure, mmHg, mean (SD)*	121.8 (14.9)	130.3 (10.8)	<.001
Diastolic blood pressure, mmHg, mean (SD)**	77.1 (9.1)	81.6 (6.9)	<.001
ASCVD, n (%)	4,555 (4.2%)	2,307 (8.5%)	<.001
Diabetes mellitus, n (%)***	3,194 (2.9%)	393 (1.4%)	<.001
CHF, n (%)	398 (0.4%)	165 (0.6%)	
Hypertension, n (%)	26,664 (24.4%)	7,837 (28.8%)	<.001
Race/ethnicity			<.001
White	101,530 (92.9%)	24,693 (90.6%)	
Black	2,321 (2.1%)	243 (0.9%)	
American Indian	87 (0.1%)	–	
Asian	803 (0.7%)	308 (1.1%)	
Hawaiian	34 (0.03%)	–	
Other/ unknown	2,632 (2.4%)	1,748 (6.4%)	
Multi-racial	1,852 (1.7%)	247 (0.9%)	
Ethnicity			<.001
Hispanic	1,633 (1.5%)	173 (0.6%)	
Non-Hispanic	107,626 (98.5%)	27,066 (99.4%)	
Smoking status, ever yes, n (%)	50,827 (46.5%)	14,274 (52.4%)	<.001
Family history of diabetes, n (%)	41,399 (37.9%)	7,813 (28.7%)	<.001
Family history of ASCVD, n (%)	60,467 (55.3%)	10,625 (39.0%)	<.001
Cohort, n (%)			<.001
Nurses’ Health Study	61,704 (56.5%)	–	
Nurses’ Health Study II	47,555 (43.5%)	–	
Health Professionals Follow-up Study	–	27,239 (100%)	

Abbreviations: ASCVD, atherosclerotic cardiovascular disease; BMI, body mass index; CHF, congestive heart failure; DBP, diastolic blood pressure; SBP, systolic blood pressure

* Missing SBP for 14,021 (10.3%) individuals

** Missing DBP for 14,139 (10.4%) individuals

*** All categories of diabetes were included

**Table 2:** Quartiles of Cumulative Excess BMI Exposure in 1990-1999.

Quartile	Lower Boundary (inclusive)	Upper Boundary (not inclusive)	Median	Interquartile Range
1	0	0.72	0.22	0.08 - 0.43
2	0.72	2.46	1.47	1.06 - 1.94
3	2.46	5.58	3.74	3.05 - 4.58
4	5.58	47.7	8.73	6.89 - 11.84

Cumulative excess BMI exposure quartiles were calculated for the entire study population (women and men). Numbers in the table represent annualized cumulative excess BMI in kg/m^2^ per year.

### Cumulative excess BMI and cardiovascular events

**Fig 3:**
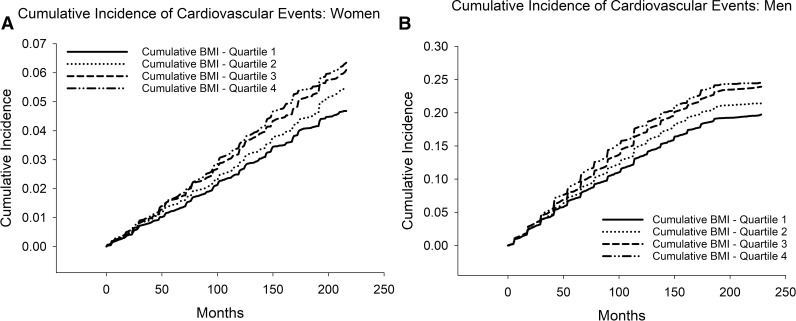
Cumulative Incidence of the Composite Primary Outcome. **A.** Women. Differences between strata were statistically significant (P < 0.001). Cumulative excess BMI exposure quartiles were calculated for the entire study population (women and men). Last 10% of the population in each group were not included in the plot due to low participant numbers and resulting wide confidence intervals. **B.** Men. Differences between strata were statistically significant (P < 0.001). Cumulative excess BMI exposure quartiles were calculated for the entire study population (women and men). Last 10% of the population in each group were not included in the plot due to low participant numbers and resulting wide confidence intervals.

Over a median of 16.7 (IQR 12.5–17.8) years of follow-up beginning in 2000, 12,048 (8.8%) of study participants experienced cardiovascular events, including 5,910 (4.3%) who had a non-fatal MI; 6,157 (4.5%) who had a non-fatal CVA and 1,719 (1.3%) who had a fatal cardiovascular event. In univariate analysis (Fig 3 and Table 3), 10-year incidence of the primary outcome ranged from 2.6% (95% CI 2.4–2.8%) for the 1^st^ quartile to 3.5% (95% CI 3.3–3.8%) for the 4^th^ quartile of cumulative excess BMI exposure for women (P < 0.001), and from 13.5% (95% CI 12.7–14.2%) for the 1^st^ quartile to 18.0% (95% CI 16.7–19.3%) for the 4^th^ quartile for men (P < 0.001).

**Table 3:** Cumulative Incidence of the Composite Primary Outcome at 10 years.

Sex	Cumulative BMI Exposure Quartile	Cumulative Incidence of Cardiovascular Events at 10 years	
		Estimate, %	95% CI
Women	1	2.62	2.43–2.82
	2	2.89	2.69–3.11
	3	3.35	3.14–3.58
	4	3.53	3.33–3.75
Men	1	13.45	12.69–14.23
	2	15.09	14.34–15.86
	3	16.80	15.96–17.65
	4	17.99	16.73–19.29

**Fig 4:**
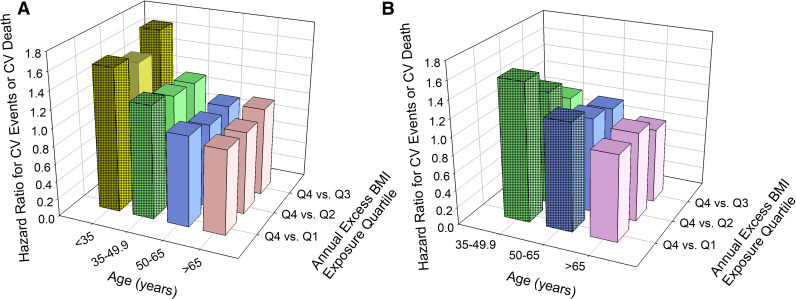
Cumulative BMI Exposure and Primary Outcome: Multivariable Analysis. **A.** Women. **B.** Men. Bars represent hazard ratios for the relationship between cumulative exposure to BMI over 25 kg/m2 between 1990−1999 and MACE-3 composite primary outcome events beginning in 2000. Cox proportional hazards models were stratified by age. Fourth quartile of cumulative exposure to BMI over 25 kg/m2 served as the reference category. Stippled bars represent associations with a P < 0.05.

In a multivariable analysis adjusted for participants’ demographics, baseline BMI and comorbidities, baseline BMI was not associated with an increased CV risk in any sex/ age subgroup when cumulative excess BMI was included in the model. Having annualized cumulative excess BMI in the 4^th^ vs. 1^st^ quartiles was associated (Fig 4) with an increased CV risk for women aged (in 1990) < 35 (HR 1.60; 95% CI 1.05 to 2.44) and 35.0–49.9 years (HR 1.27; 95% CI 1.01–1.58); and for men aged 35.0–49.9 (HR 1.57; 95% CI 1.22 to 2.03) and 50.0–64.9 (HR 1.23; 95% CI 1.02 to 1.48). There was no data available for men younger than 35. There was no difference in CV risk between 1^st^ and 4^th^ quartiles of excess BMI exposure for women 50 and older and for men 65 and older.

## Discussion

In this study of a large, nationwide, prospectively collected dataset that included participants both with and without baseline ASCVD we found that exposure to excess weight over time was a stronger predictor of cardiovascular risk than a person’s weight at a single point in time (at study entry). In the setting of the ongoing epidemic of obesity in the U.S. and worldwide, these findings have significant implications for patient care and public health.

Obesity is thought to be a substantial contributor to overall risk of mortality; and could be an important factor behind slowing or reversing growth in life expectancy [21–23]. Cardiovascular events – myocardial infarction and stroke – are together the most common cause of death in the U.S. and across the world [11,24,25]. Therefore, understanding the role of obesity in development of these adverse outcomes is vital to achieving further improvements in quality of life and life expectancy.

The present study demonstrated a strong relationship between long-term exposure to excess weight and cardiovascular risk, while the association of cardiovascular risk with baseline BMI was no longer statistically significant once long-term excess weight exposure was included in the model. From the perspective of public health, this finding can be viewed as a glass half-full. It suggests that, for a given individual, presence of obesity at a particular point in their life is not the final sentence, but a call to action. If that individual decreases their weight and thus reduces their long-term excess weight exposure, their cardiovascular risk might decrease. While few of this study’s participants were able to achieve a significant weight reduction during the excess weight exposure assessment period in the 1990s, the progress of medicine since then has made available a range of safe and effective options for weight loss, ranging from lifestyle changes to pharmacotherapy to metabolic/ bariatric surgery [26–29]. These evidence-based interventions provide a potentially life-saving opportunity for healthcare professionals to help their patients reduce their excess weight and decrease their risk of myocardial infarction and stroke. In fact, several weight-loss interventions have already been shown to reduce the risk of range of adverse cardiovascular events [30,31].

The increased cardiovascular risk from exposure to excess weight was predominantly found among younger individuals. This is consistent with other previously published studies on the relationship between exposure to excess weight and incidence of long-term complications of obesity. Recalde et al. showed that age of onset of overweight and obesity was inversely associated with the risk of 18 cancers [32] while Luo et al. found a similar relationship between the age of onset of obesity and the subsequent incidence of type 2 diabetes [33]. The inverse relationship between age of onset of a cardiometabolic risk factor and incidence of adverse outcomes is not limited to obesity: Domanski et al. demonstrated that cumulative exposure to LDL cholesterol earlier in life was associated with greater cardiovascular risk than later exposure [12]. One possible mechanism for these findings is that excess adiposity during young age may drive a higher risk of insulin resistance and development of metabolic syndrome than obesity during older age [34,35]. Further research is needed to understand biochemical and physiologic mechanisms that result in greater adverse impact of cardiometabolic risk factors in younger individuals. In the meanwhile, the apparent clinical implications are that obesity is most dangerous at younger ages and should be treated as early as possible.

Findings of previously published studies that investigated the relationship between individuals’ weight trends over time and cardiovascular outcomes have varied. Raffield et al. did not find an increased risk of cardiovascular disease among individuals whose adiposity increased during the 12-year period of observation [13]. However, 75% of participants in that analysis were over 49 years old – an age at which the effects of longitudinal excess BMI exposure were also lower or undetectable in the present study. Furthermore, the analysis by Raffield et al. had significant methodological differences from the one presented here, focusing on the direction of change rather than on the cumulative exposure to excess adiposity, possibly also accounting for the differences in the observed results. On the other hand, several smaller, studies (CARDIA, Framingham Cohort Study and EPITeen Cohort Study) have all identified trends for an increased cardiometabolic risk with greater exposure to obesity, similar to the present analysis [36–38].

The present study has a number of strengths. Its large number of participants and long duration of follow-up provided statistical power that permitted detailed analysis by sex and age subgroups. Study data was collected prospectively with little missing data that minimized possible selection bias. Validated outcome ascertainment by the NHS and HPFS studies that served as the source of study data further improved the quality of the analyses.

The results of this study should be interpreted in the light of its limitations. Participants’ height and weight were self-reported. Study participants had limited socioeconomic and racial/ ethnic diversity. Further studies should be conducted to confirm that our findings apply to populations not represented in the analysis. The study population also had a significant sex imbalance, with women constituting the majority of the study participants. However, since data on women and men were analyzed separately, this imbalance should not have affected the findings of the study. BMI was used as the primary measure of overweight/ obesity in this analysis and has well established limitations in this regard [39,40]. It is likely that the study findings do not apply to individuals whose BMI does not accurately reflect their visceral and ectopic adiposity that is thought to be primarily responsible for the relationship between obesity and the risk of long-term adverse outcomes [41,42]. Finally, this was an observational analysis that cannot definitively establish causal relationships.

This study’s findings suggest that long-term exposure to excess weight plays a greater role in subsequent cardiovascular risk than weight at a single point in time. The risk associated with long-term excess weight exposure is strongest at younger ages. These results can help inform individual patients and their clinicians considering treatment of overweight and obesity as well as public health policies.

### Conflict of interest disclosures

Turchin reports consulting for Novo Nordisk and Proteomics International; and research support from Eli Lilly and Company and Novo Nordisk. Ahmad, Neff and Kan are employees and stockholders of Eli Lilly and Company. None of the other authors report any conflicts of interest.
